# PLAFOKON: a new concept for a patient-individual and intervention-specific flexible surgical platform

**DOI:** 10.1007/s00464-021-08908-x

**Published:** 2021-12-17

**Authors:** Lukas Bernhard, Roman Krumpholz, Yannick Krieger, Tobias Czempiel, Alexander Meining, Nassir Navab, Tim Lüth, Dirk Wilhelm

**Affiliations:** 1 Research Group for Minimally Invasive Interdisciplinary Therapeutic Intervention (MITI), Klinikum rechts der Isar, Technical University of Munich, Munich, Germany; 2 Institute of Micro Technology and Medical Device Technology, Department of Mechanical Engineering, Technical University of Munich, Munich, Germany; 3 Chair for Computer Aided Medical Procedures & Augmented Reality, Technical University of Munich, Munich, Germany; 4 Department of Internal Medicine II, Gastroenterology, University Hospital Würzburg, Würzburg, Germany; 5 Department of Surgery, Klinikum rechts der Isar, Munich, Germany

**Keywords:** Individualized surgery, Surgical manipulator, Operating platform, Preoperative planning, 3D printing

## Abstract

**Background:**

Research in the field of surgery is mainly driven by aiming for trauma reduction as well as for personalized treatment concepts. Beyond laparoscopy, other proposed approaches for further reduction of the therapeutic trauma have failed to achieve clinical translation, with few notable exceptions. We believe that this is mainly due to a lack of flexibility and high associated costs. We aimed at addressing these issues by developing a novel minimally invasive operating platform and a preoperative design workflow for patient-individual adaptation and cost-effective rapid manufacturing of surgical manipulators. In this article, we report on the first in-vitro cholecystectomy performed with our operating platform.

**Methods:**

The single-port overtube (SPOT) is a snake-like surgical manipulator for minimally invasive interventions. The system layout is highly flexible and can be adapted in design and dimensions for different kinds of surgery, based on patient- and disease-specific parameters. For collecting and analyzing this data, we developed a graphical user interface, which assists clinicians during the preoperative planning phase. Other major components of our operating platform include an instrument management system and a non-sterile user interface. For the trial surgery, we used a validated phantom which was further equipped with a porcine liver including the gallbladder.

**Results:**

Following our envisioned preoperative design workflow, a suitable geometry of the surgical manipulator was determined for our trial surgery and rapidly manufactured by means of 3D printing. With this setup, we successfully performed a first in-vitro cholecystectomy, which was completed in 78 min.

**Conclusions:**

By conducting the trial surgery, we demonstrated the effectiveness of our PLAFOKON operating platform. While some aspects – especially regarding usability and ergonomics – can be further optimized, the overall performance of the system is highly promising, with sufficient flexibility and strength for conducting the necessary tissue manipulations.

**Supplementary Information:**

The online version contains supplementary material available at 10.1007/s00464-021-08908-x.

Research in the field of surgery is mainly driven by aiming for trauma reduction as well as for personalized treatment concepts. Laparoscopic surgery can be regarded as the first and maybe most relevant success towards this goal and was further advanced by the concepts of single-port and robotic surgery over the last years. Unfortunately, and despite the proven advantages of laparoscopic principles [1], one failed to identify further advantages for the latter techniques, or for single port and robotic surgery respectively, when compared to conventional laparoscopy [2, 3]. In contrast, the mentioned advancements caused an increase in costs, procedure related complexity and technical demands. Especially single-port surgery is known as technically highly demanding leading to the development of dedicated training courses [4] and instrumentation [5]. Of such, the single port system SPIDER might be emphasized as a mechanical system which was designed to overcome the limitations of the small single access and which made it to the market, as compared to the many innovations that have been published during the NOTES era [6]. However, the system did not convince in daily practice, which might be due to the associated costs, but more likely due to the one-size-fits-all concept making triangulation and proper surgical instrumentation almost impossible for the majority of patients.

These developments could thus be seen as dead-end solutions, and are seen as such by critical experts, but could on the other hand be further optimized by means of proper technologies and in terms of meeting given problems in surgical care.

With the research project PLAFOKON we thus aimed at developing an innovative single port operating system (SPOT) which combines a patient and disease specific design, realized in an overnight approach by means of 3D printing, with smart supportive technologies. In this context, an AI-enabled workflow analysis (AIWA) was developed to provide a cognitive and collaborative environment, whereas a master-slave controlled instrument management system (IMS) enables the ergonomic system control by a single surgeon. In this article, we describe this concept and report our findings for the first in-vitro cholecystectomy performed with this system.

## Materials and methods

As the main component, the PLAFOKON platform features a 3D-printed monolithic single-port operating system (SPOT), for which we have already presented multiple ex-vivo and in-vivo evaluations in previous work [7–11]. Unreported so far, this system is part of a patient- and disease-specific surgical approach, which uses individualized computer-aided design concepts in combination with AI and mechatronic control. For achievement of a patient-individual surgical system, we analyze patient-, disease- and intervention-parameters, which are used as a foundation for dimensioning the manipulator geometry to achieve an optimized design tailored to the requirements of both patient and surgeon. We use our PLAFOKON Planning Software (PPS) to support surgeons in this process. The platform possesses two independent driving units, one for mechatronic control of the manipulator arms and one for control of instruments including their exchange (IMS). The AIWA system we developed [12] is a data driven surgical workflow system that was trained with clinical data from real patients. The remaining domain gap between real patients and our phantom in terms of lighting, viewing angle and phantom tissue properties prevented us from using the AIWA in this study. In the context of this study, we were especially interested in testing and exploring the technical and mechanical advancements of the PLAFOKON platform.

As described in the following, we used porcine organs (livers including gallbladder) for our trial surgery, which were procured from the abattoir. Our study did not involve human participants or animal trials, which is why written consent and IRB approval were not required.

### Single-port overtube

**Fig. 1:**
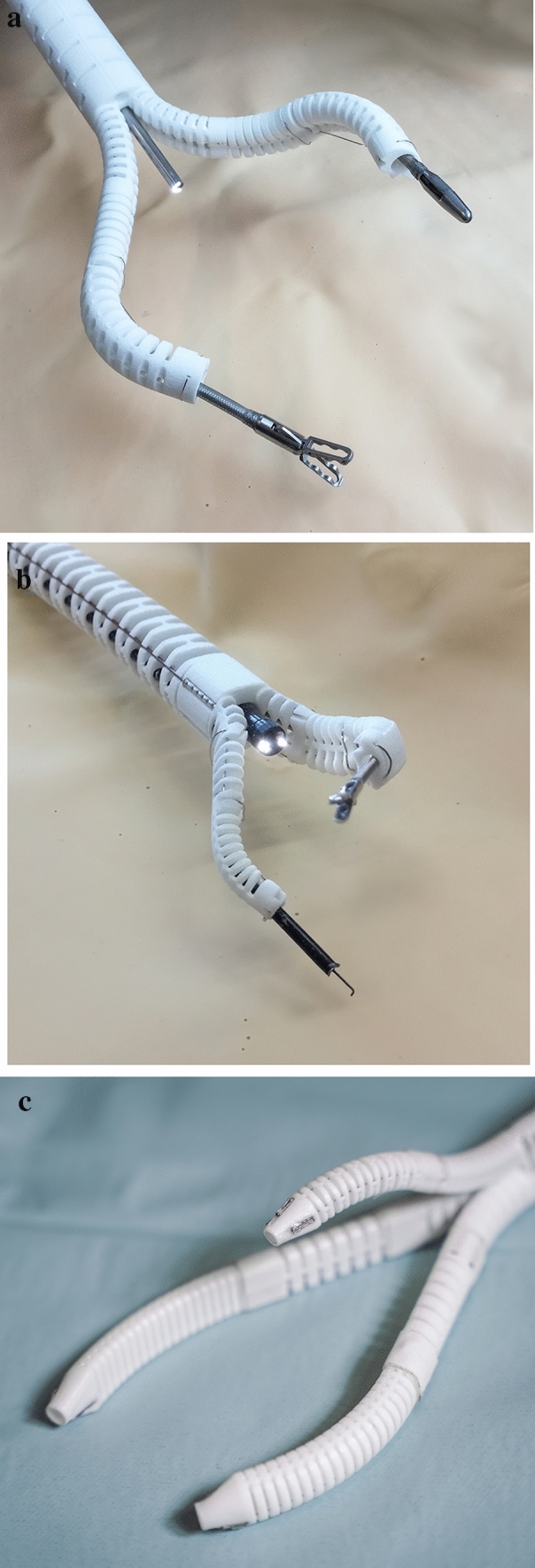
Examples of different variants of the SPOT surgical manipulator are shown. **a** two-arm configuration for single-port scenarios, **b** endoscopic overtube for NOTES scenarios, **c** three-arm configuration with actuated camera arm for single-port scenarios

The single-port overtube (SPOT) is a snake-like surgical manipulator for minimally invasive interventions. The system design is highly flexibly and can be adapted for a variety of different applications, including single-port and NOTES scenarios in visceral surgery (Fig. 1). Due to its rapid and inexpensive manufacturing process by means of 3D printing, the production of patient-individual surgical manipulators becomes feasible. This is complemented by an automated design process for the manipulator structures based on a monolithic flexure hinge design which makes assembling of the system unnecessary. Depending on the required forces and the application, the flexure hinge segments can be adapted as well as the entire shape of the manipulator. The flexure hinges are arranged in parallel for the respective degrees of freedom to allow a deflection of the manipulator arm in one plane per degree of freedom. They are designed as circular flexure hinges with adapted dimensions according to the load case. The manufacturing of SPOT is realized using selective laser sintering (SLS) with polyamide 12 (PA2200, EOS, Krailling, Germany), which is biocompatible according to EN ISO 10993–1. To improve the long-term strength of the flexure hinge structures, they are post-processed by means of surface smoothing (PostPro3D, AMT, Sheffield, UK).

In its most common form, the design of SPOT resembles known concepts and features two flexible and independently movable manipulator arms to be equipped with conventional endoscopic instruments and a flexible optic, either in form of a flexible endoscope or as a steerable arm directing an internal optic. The robot’s degrees of freedom are actuated using Bowden cables that can be connected to either a motorized or a purely mechanical control unit. The type of control unit can be chosen depending on economic and infrastructural preconditions. While a motorized configuration is beneficial regarding ergonomics and maximum manipulation force, a completely mechanical approach allows for an isolated usage of the entire system, e.g. in an emergency scenario or for remote rural areas of developing countries.

### Inference of design parameters

The geometry of the entire system as well as specific features such as the length and number of manipulator arms, the need for an auxiliary working channel, the size of the system body, and its robustness strongly depend on the intended intervention and show relevant variations when, for example, comparing a cholecystectomy (small working space, easy and directed retraction, highly standardized) and a sigmoid resection (large working space, high forces necessary for multidimensional retraction). Based on intervention-specific requirements which originate from surgeon experience and interoperative measurements, we defined basic surgery-related system requirements which are further refined and adapted on patient-specific parameters (patient size, body mass index, distance between entry point and surgical field, etc.). Patient parameters are derived from interviews, from a patient report form and from preoperative imaging (CT scan, ultrasound). In this context, exemplarily, an obese male patient who has passed an episode of cholecystitis requires a stronger and larger system design as compared to a slim young female patient who is operated for a symptomatic gall stone. Finally, several design and construction parameters were selected as relevant and compiled using our dedicated PPS software tool described in the following section.

### Preoperative planning software

For a structured management of all patient and design data, we developed the PLAFOKON planning software (PPS) to support clinicians during the preoperative planning phase. The PPS assists the surgeon in collecting, storing, analyzing, displaying and querying information on the current case as well as previously finalized cases that were treated with the PLAFOKON operating platform. Based on this, the PPS determines a suitable manipulator design and yields predictions regarding the planned intervention.

**Fig. 2:**
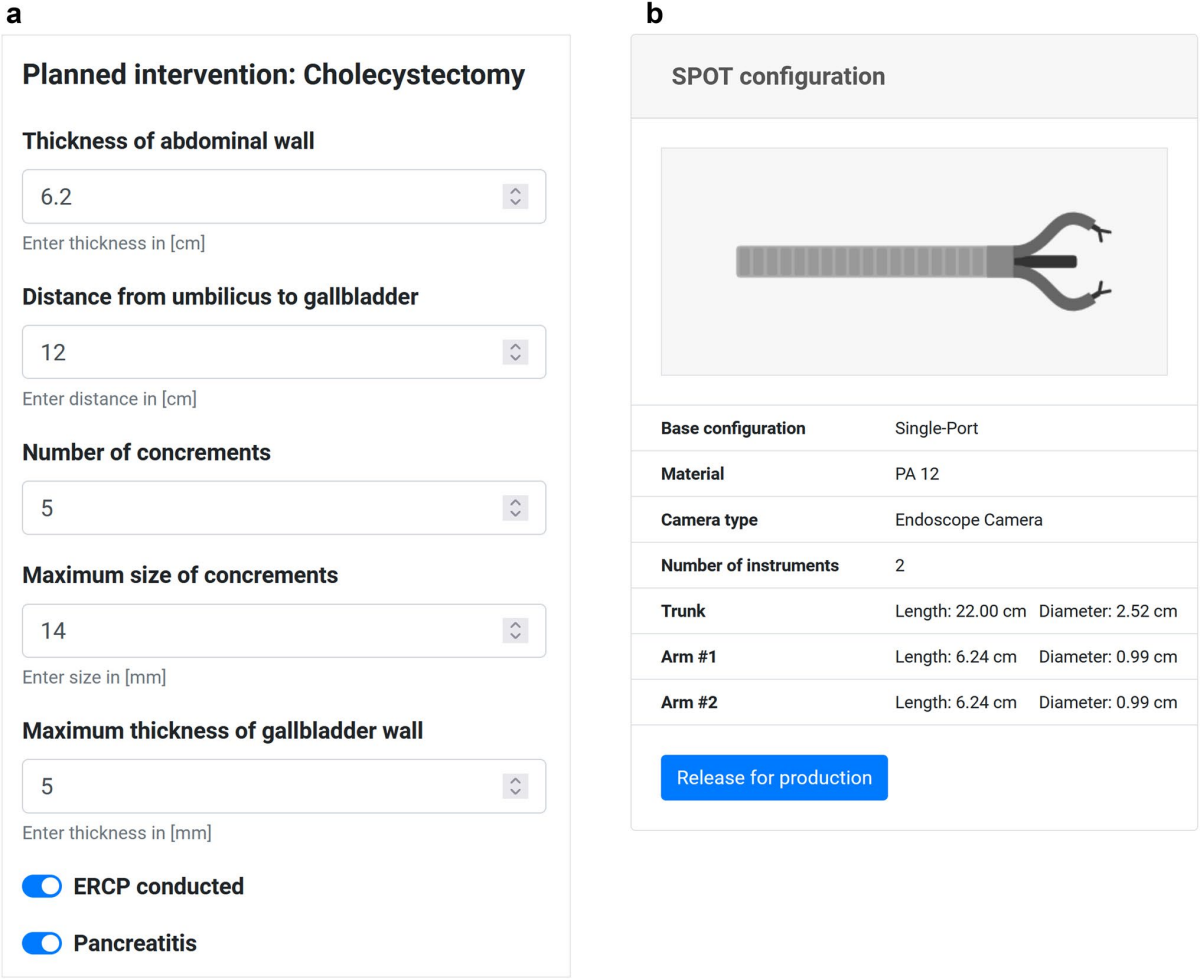
Excerpt of the graphical user interface of the PLAFOKON planning tool; **a** Parameters specific to the intervention (here: cholecystectomy); **b** Design parameters for the SPOT manipulator calculated by the planning tool

The preoperative workflow is intended as follows: The responsible physician gathers relevant information on the patient's physical condition (size, body mass index, etc.) and anatomical characteristics. This is done in the course of patient interviews and by conducting preoperative imaging. For a structured collection of all relevant information, the PPS offers a combination of input masks, queries to the clinical information system and an automated analysis of pre-operative data. The resulting data pool can be divided into general patient information and additional intervention-specific parameters. General data includes the patient's age, gender, BMI, addictive behavior, and medical history. For the cholecystectomy use case, the following procedure-specific parameters are recorded (Fig. 2a): abdominal wall thickness, distance from umbilicus to gallbladder, number and maximum size of concrements within the gallbladder, maximum thickness of the gallbladder wall, presence of a pancreatitis (inflammation of the pancreas) and conduction of an endoscopic retrograde cholangiopancreatography (ERCP).

The gathered information is processed by the PPS to provide basic design parameters of the SPOT system, such as arm length and thickness, mainly to ensure proper structural strength and operating range of the manipulator. For the cholecystectomy use case, our software calculates the shape and dimension of SPOT based on 6 patient- and 7 procedure-related parameters and visualizes the resulting optimized design within the GUI (see Fig. 2b). For subsequent validation and further optimization, we plan to implement a training mode in the future which the surgeon can use to test the manipulator within a virtual environment visualizing patient-specific anatomy and 3D SPOT geometry, e.g. to test the reach and mobility of the manipulator arms. After approval by the surgical expert, the system requirements and the computer calculated design of the manipulator are then forwarded to the production facility to manufacture the manipulator within a few hours only using the 3D printing process selective laser sintering and very few mechanical components (mainly Bowden wires).

### Experimental setup

**Fig.3:**
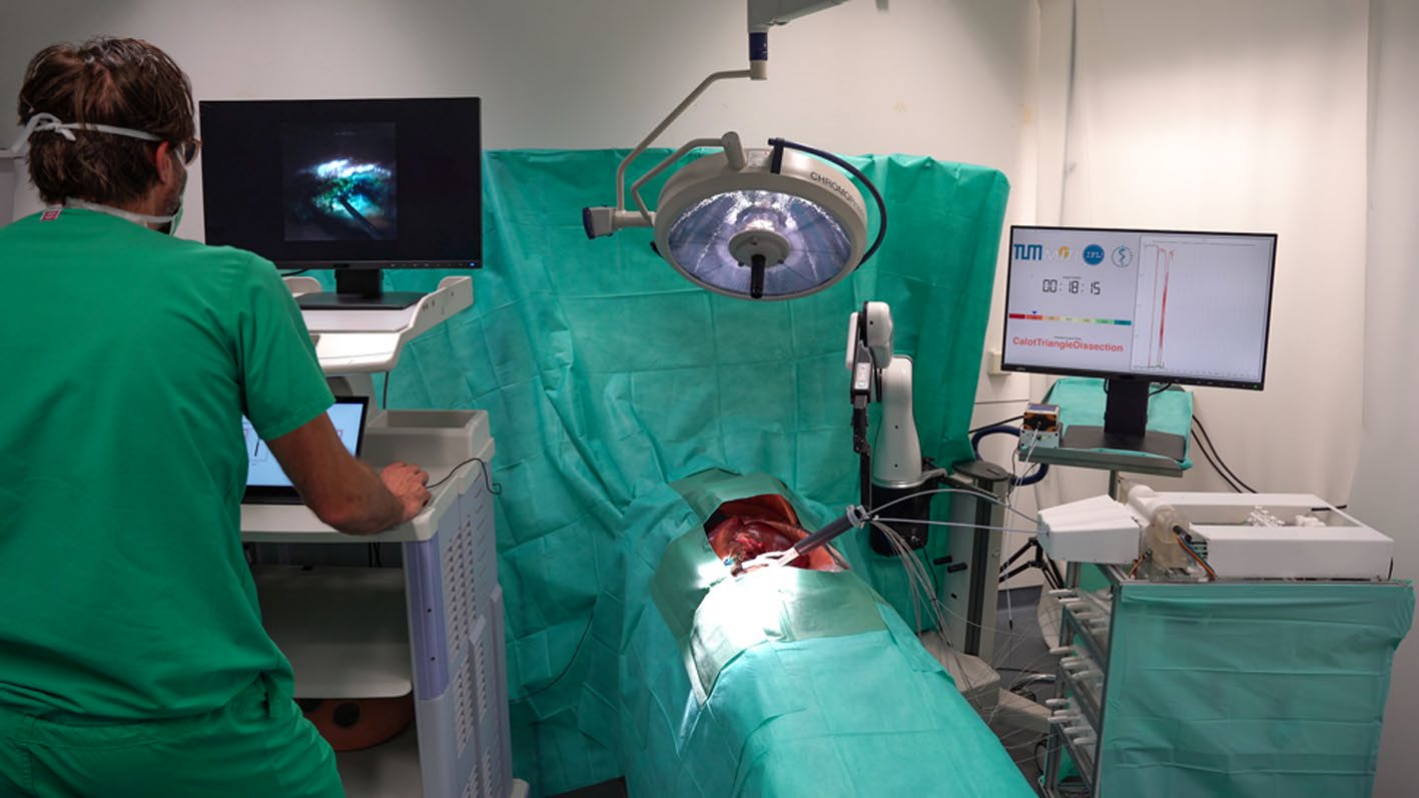
Experimental setup of the PLAFOKON operating platform, as used for conducting our trial surgery

For conducting the trial surgery, the entire PLAFOKON operating platform was set up within a mockup OR environment (see Fig. 3). As a dummy patient, the validated endoscopic/laparoscopic training phantom ELITE (CLA, Coburg, Deutschland) was placed on the OR table and equipped with a porcine liver including the gall bladder. To ensure realistic tissue behavior, the porcine livers had been freshly procured on the same day shortly before starting the trial surgery. The dimensions of this phantom and the respective distances (designated entrance site to gallbladder, midline to gallbladder, thickness of abdominal wallet) were used to calculate an optimal system layout to be used during cholecystectomy. The resulting SPOT was subsequently printed and prepared for use during this experiment.

The instrument management system, which is a central component of our operating platform, was equipped with off-the-shelf instruments for flexible endoscopy, in particular a grasper – available on the left working channel of the SPOT system – and both a hook knife and a second grasper – available on the right channel. An electrocautery device was held available to enable cutting and coagulation manipulations by means of high frequency (HF) surgery.

An endoscopic camera by the Soliton Laser- und Messtechnik GmbH (Gilching, Germany) was inserted into the camera channel of the SPOT system. For illumination, the built-in light source of the camera was used. The camera image was transmitted to the PLAFOKON console and displayed to the surgeon. For this experiment, we further used a 10 mm 30° rigid optic (Karl Storz, Tuttlingen, Germany) both for improved illumination as well as for documentation purposes.

## Results

In the following, we describe the results of our efforts to develop and evaluate a patient-specific minimally-invasive surgical platform. We begin with a description of the surgical platform and its components, which is followed by a summary of the trial surgery we performed.

### Operating platform

**Fig. 4:**
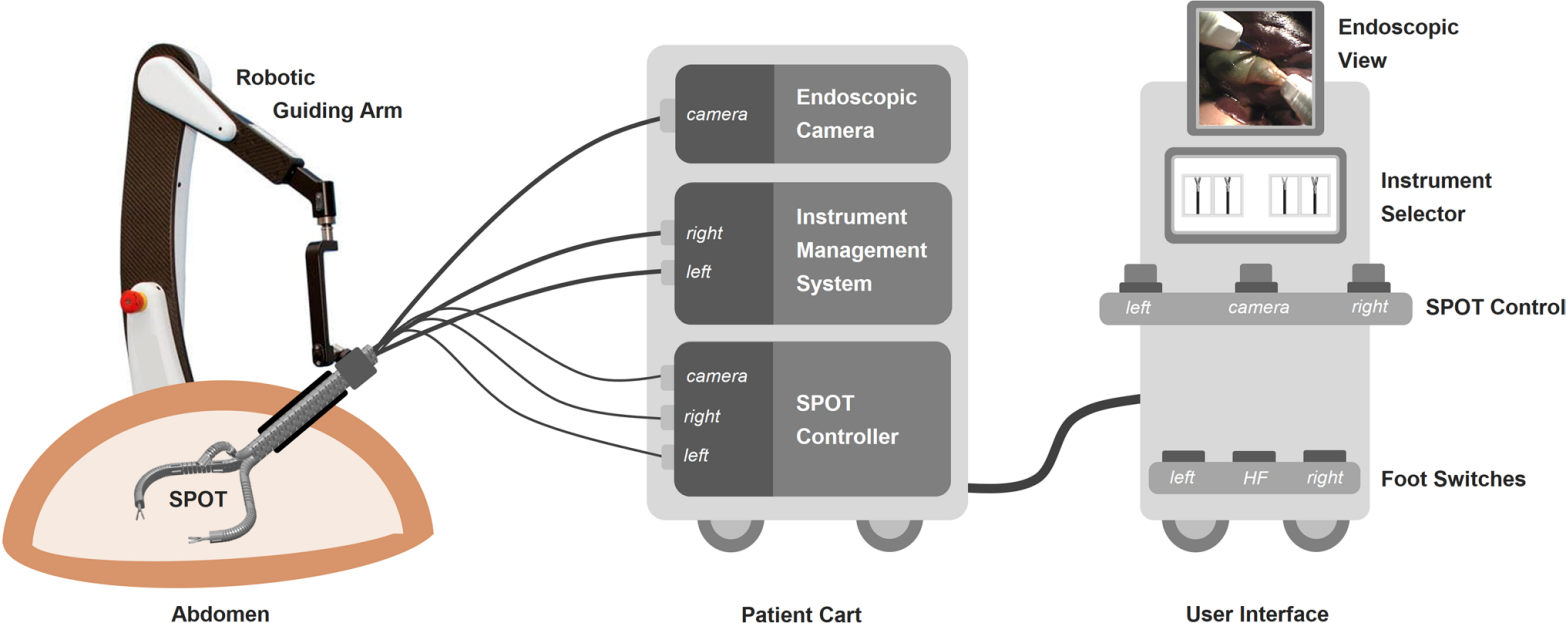
Components and layout of the PLAFOKON operating platform are shown

The components and layout of our PLAFOKON operating platform are schematically shown in Fig. 4. At the heart of the platform, the SPOT manipulator is the universal tool for performing surgical manipulations and needs to be fully controlled by the surgeon. The user interface of the platform provides an endoscopic view, an instrument selector, controls for the SPOT’s degrees of freedom, and foot switches for actuating the instrument tips as well as for applying electrocauterization. A patient cart contains all mechatronic control components necessary for actuating the manipulator. This includes the SPOT controller, which is receiving the user input and actuates the Bowden wires accordingly, and the instrument management system (IMS) responsible for inserting, actuating and retracting endoscopic instruments inside of SPOT’s two working channels. The patient cart also contains the endoscopic camera, which is inserted into SPOT’s camera channel.

**Fig.5:**
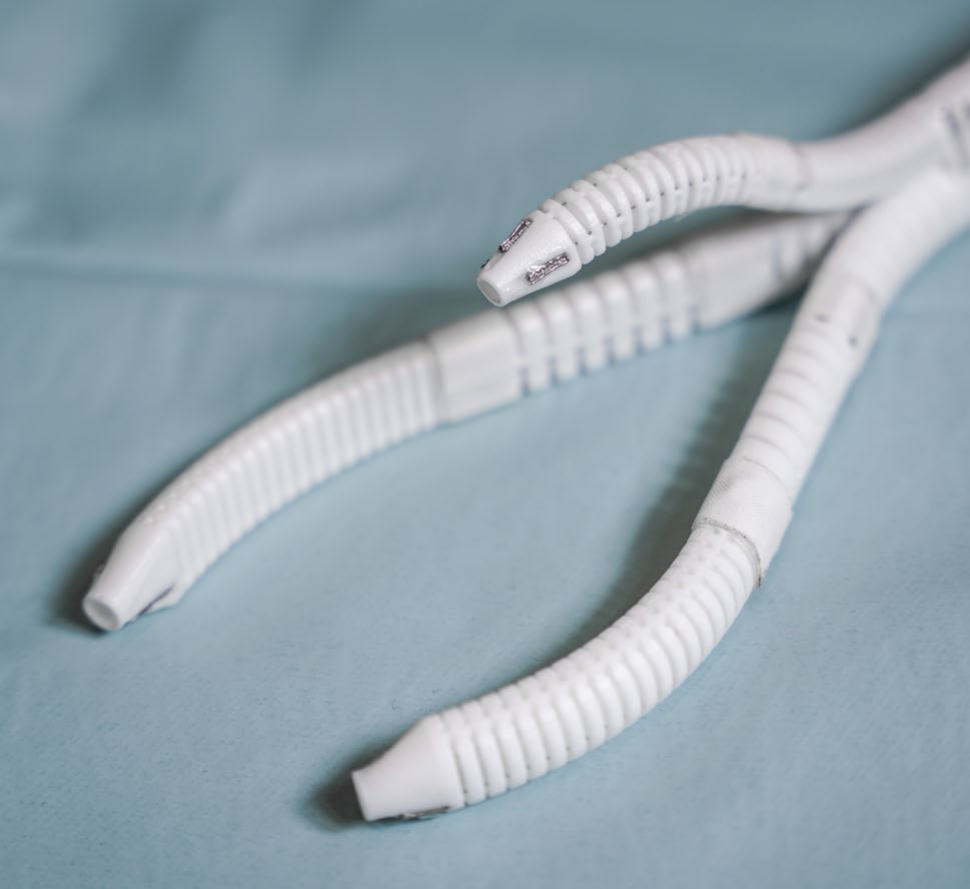
The manufactured SPOT prototype used for trial surgery is shown

**Fig. 6:**
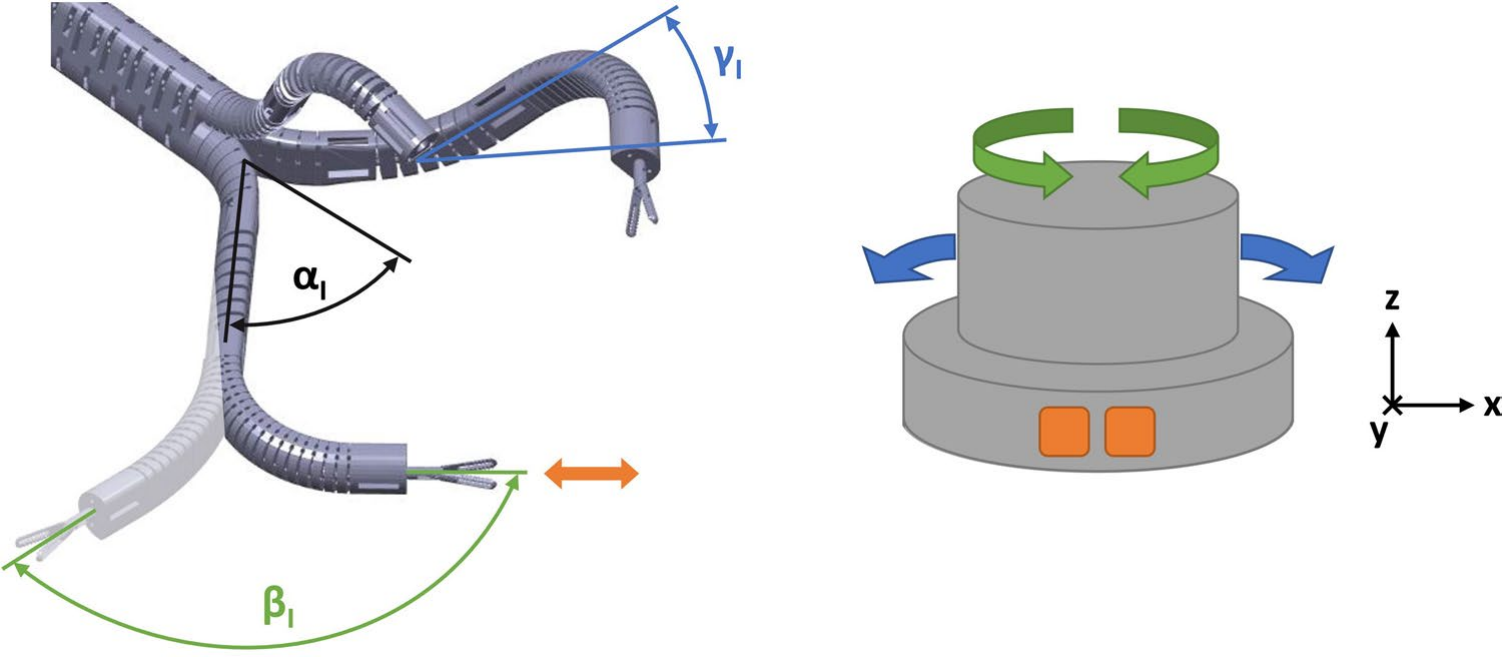
This figure illustrates the degrees of freedom (DOF) of the SPOT instrument arms and how these can be controlled using space mouse devices. Rotation around the z-axis moves the instrument tip to the left or to the right (${\beta }_{I}$). Rotation around the y-axis moves the instrument tip up or down (${\gamma }_{I}$). Using the function buttons of the space mouse, the instrument tip can be moved forward or backward

For the trial surgery presented in this paper, the available data on the training model and the porcine gallbladder resulted in a 3-armed SPOT variant with two manipulator arms of identical length and one camera arm (see Fig. 5). The system was designed in a single-port configuration and a motorized control approach was chosen for actuating the end effectors. The degrees of freedom (DOF) of the instrument arms are shown in Fig. 6. For our prototype, the following maximum values are achievable: ${\alpha }_{I}=35^\circ , {\beta }_{I}=240^\circ , {\gamma }_{I}=\pm 90^\circ$. After inserting the SPOT robot into the abdomen, the ${\alpha }_{I}$ DOF is automatically moved into a fixed position ($35^\circ$) by the SPOT controller to achieve triangulation. Subsequently, DOF ${\beta }_{I}$ and ${\gamma }_{I}$ can be manipulated by the surgeon within the ranges given. The DOF of the camera arm can be defined accordingly, with the following maximum values achievable with our prototype: ${\alpha }_{C}=55^\circ$ (fixed vertical abduction necessary for “swan’s neck” pose), ${\beta }_{C}=150^\circ$ (variable vertical working range), ${\gamma }_{C}=\pm 85^\circ$ (variable horizontal working range). The lengths, thicknesses and inner diameters of the prototype are described by Table 1. A third working channel with a diameter of 5 mm was integrated into the shaft, which can be used for the manual insertion of instruments. Manipulation forces and repetition accuracies under load have been studied in previous work [7].

**Table1:** Lengths, thicknesses and inner diameters of the SPOT prototype used for the trial surgery

	length [mm]	max. thickness [mm]	inner diameter [mm]
instrument arms	160	15	5
camera arm	95	10	4
shaft	220	24	-

**Fig. 7:**
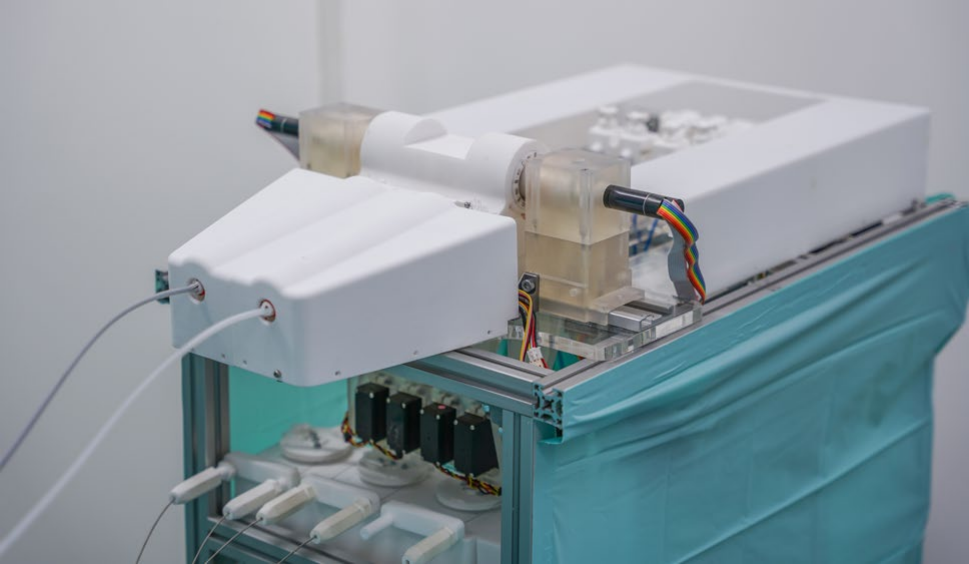
The instrument management system used for inserting, actuating and retracting endoscopic instruments inside of SPOT’s working channels is shown

Since the final tissue interaction is not only achieved by deflecting the manipulator arms themselves, but also by moving and actuating the endoscopic instruments inside of the arms, a component for handling the instruments was necessary. We realized this in form of an instrument management system (IMS, see Fig. 7), which stores, changes and actuates up to four different endoscopic instruments. On demand, the IMS automatically retracts one of the currently inserted instruments from the SPOT system and, according to the needs of the surgeon, inserts a different instrument into the channel. Insertion and retraction are achieved by a mechanism based on rotating cylinders, which are pressed on the instrument from both sides and transport the instrument via friction forces. Once the formerly inserted instrument is completely retracted, the new instrument is inserted into the working channel by reverting the direction of cylinder rotation. After an instrument has been inserted, the IMS provides fine control over how far the instrument’s tip is protruding from the manipulator’s arm. This allows for a certain range in z-direction to access anatomic structures in front of the manipulator and allows for pulling or pushing tissue by the surgeon. The end effectors of the endoscopic instruments are controlled independently by the IMS using the designated mechanics provided in the handpieces of each instrument.

In a telemanipulation-like fashion, the entire PLAFOKON system is controlled remotely using a user interface (surgeon console) located in the non-sterile area of the OR environment. Therefore, the surgeon is not required to be sterilely washed while operating (master-slave-design). The user interface provides bundled access to all functionalities of the PLAFOKON system, i.e. the SPOT manipulator, the endoscopic instruments and the endoscopic camera. With a 3D mouse for each of the surgeon's two hands, the arms of the SPOT manipulator can be moved independently (see Fig. 6). The insertion distance of each instrument can be adjusted to the surgeon’s needs via the lateral keys of the corresponding mouse. Using another 3D mouse, the camera arm of the SPOT manipulator can be controlled to adjust the viewing direction of the endoscopic camera. After changing the viewing direction, the setting can be fixed in place so that the surgeon can remove the hand from the mouse again. To request a change of instruments, the desired instrument can be selected using a touch-based graphical user interface. As explained before, the IMS will handle the retrieval of the current instrument and the insertion of the desired one.

The SPOT manipulator is guided and held in place by the robotic arm SOLOASSIST II (AKTORmed GmbH, Barbing, Germany) which, within a certain range, allows for changing the insertion angle of the single-port system. This may be necessary during surgery to gain easier access to different parts of the surgical site. Via touch-up, the pivot point of the single-port access can be programmed into the SOLOASSIST robot. After that, the surgeon can rotate the SOLOASSIST’s end effector – and thus the SPOT manipulator – around this point to effectively change the working direction of the entire system. The SOLOASSIST robot can also be used to insert the SPOT manipulator deeper into the patient’s abdomen or retract it.

In case of an adverse event during surgery – e.g. due to an unexpected bleeding that cannot be controlled in a minimally invasive manner – the surgeon might choose to switch to a conventional, open access. In this case, the entire operating theatre needs to be transformed rapidly, which we refer to as emergency retrieval. The command for starting this process is given by the surgeon using the graphical user interface. In an emergency event, the endoscopic instruments are first closed and retracted from their working channels. The end effector arms of the SPOT system are then straightened, and the entire manipulator is removed from the port – and thus from the patient's abdomen – with the help of the SOLOASSIST robot. Both the SOLOASSIST and the IMS can then be manually moved out of the way to make space for open surgery.

### Trial surgery

The trial surgery was performed by a senior surgeon affiliated with the department of surgery at Klinikum rechts der Isar (Technical University of Munich, Germany). While the surgeon was trained and highly experienced in robotic laparoscopic interventions using the Da Vinci robot, he was performing surgery with the PLAFOKON system for the first time. In the following section we report on the chosen line-of-action and the steps that were required for performing the cholecystectomy with our system.

**Fig. 8:**
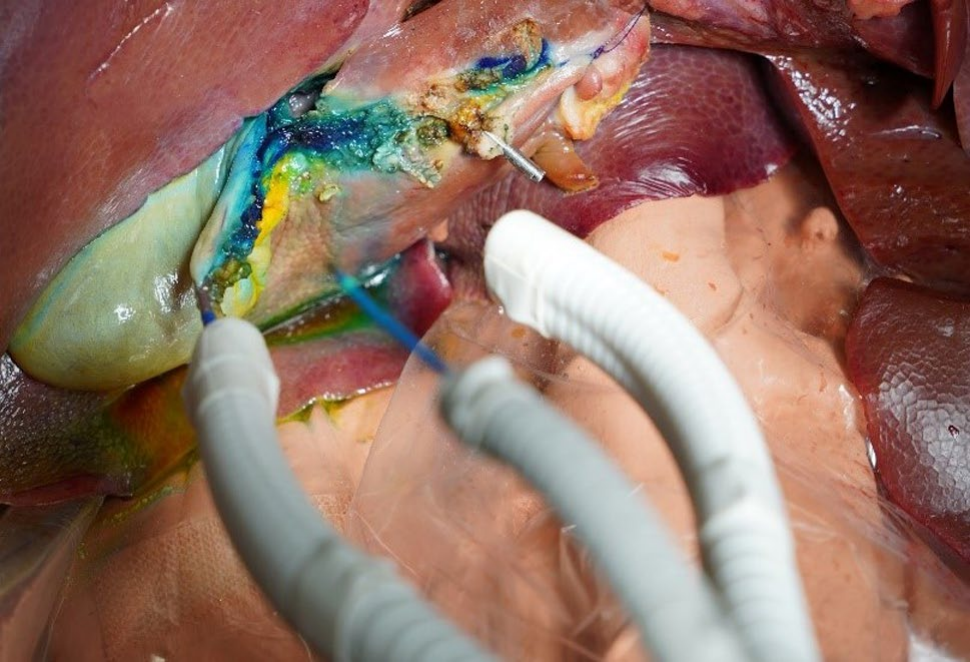
Dissection of the gallbladder during our trial surgery

In accordance with the usual procedure for minimally invasive cholecystectomy, port placement was followed by preparation of Calot's triangle. Applying the principle of triangulation, an atraumatic gripper in the left working channel was used to pull the gallbladder neck in lateral direction and create pretension on Calot’s triangle. The cystic duct and artery were exposed by blunt dissection with an endoscopic forceps on the right working channel, while deliberately applying monopolar HF coagulation. The vessels were closed using an endoscopic clip applicator, which, due to its very limited application during the intervention, was not provided by the IMS but brought in via the third working channel. Afterwards, a change of instruments was performed by the IMS to exchange the forceps for a hook knife on the right working channel. The hook knife was used as primary tool throughout the subsequent cutting and dissection phases of the cholecystectomy. Cutting was achieved by pulling the infindibulum of the gall bladder towards the manipulator – to separate them from the liver bed – using the left end effector, while performing downward movements from ventral to dorsal using the hook knife and HF cutting. A similar approach was used for the actual dissection of the gallbladder: While preloading the gallbladder neck using the left-hand gripper, the adhesive tissue was dissected and simultaneously coagulated by up and down movements of the hook knife (see Fig. 8). During that, in accordance with standard laparoscopic practice, we attempted to target the line of highest tension. Additional support during dissection was achieved by injection of blue-stained water into the adhesive layer for slight separation of the gallbladder by formation of a cushion.

With the complete resection of the gallbladder, the procedure was finalized after 78 min.

## Discussion

The successful removal of the porcine gallbladder in our phantom setup demonstrates the feasibility of our concepts. Overall, we can report that the design of our prototype fulfilled the essential requirements regarding working space and force application. Despite the prototypical design of the system, targeted and effective manipulation of the surgical instruments was possible, and the intervention was completed within an acceptable time range. Nonetheless, there were some important lessons learned regarding the SPOT manipulator and peripheral modules of the PLAFOKON operating platform, which we will discuss in the following.

### Working space

For the most part, the freedom of movement of the robotic arms was sufficient and, in conjunction with the additional degrees of freedom provided by the SOLOASSIST robot, allowed for flexibility throughout the intervention. However, we found that our choice of port placement and the angulation of the SPOT manipulator to the field of interest can be improved. As usual and according to surgical practice, we chose the navel for insertion, while the orientation of the surgical manipulator was focused on the main and central area of the intervention, for which the best exposure and instrumentation quality was anticipated. Accordingly, we inserted and fixed the SPOT system in a straight line and direction to Calot’s triangle. While this approach worked perfectly throughout the preparation, clipping, and cutting phases of the cholecystectomy, we experienced difficulties during the dissection phase, where we had to reorient the manipulator to face more laterally towards the gallbladder body. Due to this, the right instrument was getting to close to the liver surface, which forced us to retract the entire manipulator by a few centimeters. To counter this, we advanced the left instrument further out of the working channel to be able to reach the gallbladder. However, with the instrument moved out, instrument stability decreased noticeably due to the missing guidance by the manipulator arm. This was problematic when pulling the gallbladder laterally in order to create the necessary preload required for cutting through the tissue. Furthermore, the additional length of the instrument affected the amount of deflection at the tip, since the effective lever arm was increased. Accordingly, rather small movements of the joint resulted in considerable amplitudes of the tip, which was detrimental to precise manipulating and increased the total duration of the procedure.

We believe that the issue described in the previous could be mitigated by an asymmetric design of the manipulator, where one arm is longer than the other one. Additionally, placement of the port can be optimized, in this case slightly towards lateral and distal, such that the instruments do not need to be extended considerably to reach the lateral part of the surgical site. Therefore, we argue that the port placement should become an essential aspect during the patient-individual design of the SPOT system since it has a significant impact on the manipulator’s geometry and its intraoperative versatility.

Furthermore, our current prototype could have limitations when it comes to the exposure of the gallbladder, at least in humans. While we did not experience any issues during our ex-vivo trials, two instrument arms might not always be enough in this regard. However, due to the adaptability of our approach, it is easily possible to include a third instrument arm into the SPOT system whenever needed. This would enable the surgeon to expose the gallbladder in a similar manner as commonly done in standard laparoscopic approaches.

### Endoscopic view

Furthermore, limitations regarding the endoscopic view were perceived as a major impediment by the surgeon, mainly throughout the dissection of the gallbladder. In part, this was due to the inferior low-resolution video signal provided by the flexible micro-optic (1 mm), which made it difficult to clearly identify the dissection layer. However, this issue can easily be solved by exchanging the camera we used for a model with better image quality and illumination. A further impediment to the endoscopic view was due to occlusions introduced by the manipulator arms themselves. This prevented a direct view on the instrument tips in some situations, which then made precise manipulation of the tissue difficult. By thinning down the manipulator arms towards the instrument tip, this problem was significantly improved in an updated version of the SPOT system. Lastly, having to guide the camera manually was perceived as a distraction to the surgical workflow, since the tissue manipulation is interrupted each time the camera needs to be repositioned. Consequently, a semi-automated camera guidance mechanism is desirable. Subsequent to the experiment described in this paper, we therefore implemented a camera tracking mode in which the camera arm automatically follows one of the instrument arms. Which arm should have priority can be selected using the GUI. Another possibility is to detect anatomical structures of interest and direct the camera to these locations accordingly. Such a recognition can be realized by applying modern AI algorithms, even when running cloudless on embedded systems, as we were able to demonstrate in past work [13].

### Control interface

As explained before, system control was achieved by means of two space mouse devices, one for each side, which allowed for control of the SPOT system and the instruments in site, a touch screen that enabled switching from one instrument to the other, and foot pedals to open and close the instrument tips and to apply HF current. Although this set-up was provisional with only a lose placement of controls, it did yet allow for intuitive and precise movements of the system and mainly contributed to the successful resection of the gallbladder. With the exception of the clip applicator, which, as of yet, has to be operated manually, the current configuration already allows for performing single surgeon procedures and thus could help to compensate for staff shortages.

The way we mapped the degrees of freedom of each space mouse to the corresponding end effector turned out to be highly intuitive for the surgeon and minimized the learning effort at the beginning of the intervention. The fact that resetting the range of motion is not required (contrary to, e.g., the da Vinci system), was also perceived as beneficial by the surgeon. However, the displacement range of space mouses is rather limited. When mapping this short displacement to the wide range of motion of the manipulator arms, precision may suffer, especially when the instrument tip is advanced far out of the working channel and the lever arm increases. The integrated spring-based mechanical reset of each degree of freedom was also identified to be suboptimal, since this causes the manipulator to be restored to its neutral position, every time the surgeon’s hands are released.

While the overall performance of our control interface was promising, we aim at designing a dedicated cockpit or console in the future. This would enable the surgeon to operate in a sitting position and would offer fixed and maximally ergonomic placement of the manual controls and the foot pedals.

## Concluding remarks

In this paper, we presented the PLAFOKON operating platform and the peri-operative workflow support concepts associated with it. As a central component of the platform, the SPOT surgical manipulator has been presented, which is a snake-like surgical robot for NOTES and Single-Port interventions. We described our approaches and implementations for realizing a patient-individual design process of this robot, in particular using an intuitive planning software and rapid manufacturing techniques by means of 3D printing. This can be considered a groundwork for patient-specific provision of surgical manipulators that does not fail to fulfill real-world constraints regarding time, costs, and accessibility.

We demonstrated the effectiveness of our operating platform by conducting the first in-vitro cholecystectomy performed with this system. While some parameters – especially regarding usability and ergonomics – can be further optimized, the overall performance of the system was highly promising, with sufficient flexibility and strength for conducting the necessary tissue manipulations.

Now that the fundamental technical puzzle pieces for patient-specific minimally invasive surgical therapy are in place, we aim at systematically investigating the relationships between patient anatomy and optimal manipulator design in future work. In particular, this includes the consideration of different surgical procedures and the unique requirements associated with them, both in terms of access, working space, and the forces to be applied.

## Supplementary Information

Below is the link to the electronic supplementary material.

Supplementary file1 (MP4 77228 KB)
